# Book Reviews

**DOI:** 10.1038/sj.bjc.6690211

**Published:** 1999-03


*British Journal of Cancer* (1999) **79**(7/8), 1315–1317
© 1999 Cancer Research Campaign
Article no. bjoc.1998.0211

### Book Reviews

### When cells die – a comprehensive evaluation of apoptosis and programmed cell death

Edited by Richard A Lockshin, Zahra Zakeri and Jonathan L Tilly: Publication details, pp. 504, £65, ISBN 0–471–16569–7. Wiley-Liss: New York, 1998

‘When cells die…’ is a collection of reviews written by several different international researchers, from disciplines including cell and developmental biology, neuroscience, immunology, cancer research and virology. It groups these varied views on cell death into five sections: the phenomenon of cell death, themes and approaches to cell death, cell death when mitosis is high and evanescence is desirable, cell death in long-lived cells and the clinical relevance of apoptosis. The total of seventeen chapters cover a range of topics including the evolutionary origin of programmed cell death, caspases, cell death in plants, apoptotic signalling in lymphocytes, and importance in cancer therapy.

A substantial book with high aspirations, it aims to inspire those not yet captive of the field to evaluate the meaning and impact of cell death in their own particular discipline. It targets both the mature student and scientist, and addresses a great diversity of themes which unfortunately lack a unifying thread. The reader is presented with a host of different definitions of terms related to cell death, referring to very similar morphological concepts. This, perhaps, would deter a novice. It is also clear from the sometimes contradictory accounts by various authors that there are considerable and fundamental difficulties related to studying dying cells. There is no mention of the newer experimental candidates such as Annexin V and clusterin, but this is probably just a reflection of the fast-moving nature of the field.

In general, the reviews are erudite and written in an interesting variety of styles. All attempt to address the relevance of cell death to their work and are relatively up-to-date, current to 1997. However, most are hampered by lack of illustrations. For example, long descriptions of life cycles of experimental plant and animal subjects, or signal transduction pathways could have been simplified by the use of diagrams or models. A notable exception is the review of genetic approaches to programmed cell death in the nematode *Caenorhabditis elegans*, which is well written and not broken up, unlike certain others, by long lists of cross-references.

This book is probably most suitable for those who have a good understanding of cell death, rather than for those who had previously avoided the field. Its strength lies in the global view it provides in one source. It is more a selection of accounts rather than a comprehensive evaluation – possibly an unrealistic ideal, considering the range of disciplines covered.

Finally, the book did reach its stated aim of making me evaluate my work, by introducing to me the term ‘retrorecognition’ in its first chapter. This is the phenomenon by which scientific or other anomalies are not recognized until they have been given an explanation in a new conceptual framework (Lightman and Gingerich, 1991). Having produced several anomalies in my own scientific research, I have realized it is just frameworks that I am seeking.

*Dr Kathreena M Kurian*

### REFERENCE

Lightman A and Gingerich O (1991) When do anomalies begin? *Science* **255**: 690–695

### Clinical Trials in Oncology

Edited by Green, Bendetti and Crawley: Publication details, £45, ISBN 412–996316. Chapman and Hall: London

*Clinical Trials in Oncology* is based entirely on the experience of the Southwest Oncology Group statistical centre, Seattle, Washington, USA. All the examples are taken from trials conducted by the Southwest Oncology Group (SWOG) over the past 40 years.

Over this period, it is clear that the development of methodologies, policies and protocols have led to the present robust and well-defined guidelines on how clinical trials in oncology should be organized.

This book should be read by anyone who is thinking of setting up a trials group, be it for single-centre or for multicentre studies. More specifically, it should be read by anyone who is thinking of entering patients into trials organized by a trials group. Equally, medical staff already involved in entering patients into trials will benefit from reading the sections of the book relevant to them.

The book is not mathematical, it is almost entirely descriptive, and its examples illustrate good and bad aspects of past trial designs. There are many references to the more technical aspects for those who need to know more.

After a brief introductory chapter, there is a chapter on statistical concepts. This can best be summarized by the last paragraph: ‘This chapter has introduced key statistical concepts and analyses. Understanding the basics will help in understanding why statisticians choose specific designs and analyses in specific settings. These choices are the subject of the rest of the book.’

The third chapter covers the design of clinical trials. It briefly discusses phase I trials and goes into more detail on phase II trials, including the standard SWOG phase II trial design. It finishes with phase III trial methodology and emphasizes the importance of randomization. Throughout, it states the need for well-defined end points.

Chapter 4 discusses multiarm trials, that is when there are more than two treatment arms. The take-home message here is to ask simple questions (compare just two arms) and get straightforward answers, as opposed to asking many questions and running the risk of unclear answers, especially if the sample size is not adequate for the multiarm setting.

The next few chapters cover the problems and requirements of clinical trial management. In chapter 6, they discuss data management and quality control. They put forward the standard SWOG protocol design. This should be read by all parties who are thinking of setting up a multicentre clinical trial. Hopefully, they will then appreciate the work involved and the disciplines involved in organizing a successful trial. For those wishing to take part in entering cases into trials, this should give them an insight into what they are letting themselves in for!

The other two chapters in this section cover problems, pitfalls and dilemmas of interim analysis, data monitoring committees and reporting of results. These three chapters taken together cover the many aspects of organizing clinical trials, whether they are single or multicentre.

Chapter 8 covers the pitfalls of trying to overinterpret the results from trials or studies. There is a section on using historical controls and the inherent problems involved. Among other topics discussed are dose intensity analysis and subset analyses. They highlight what can go wrong.

The penultimate chapter is on exploratory analysis and the identification of prognostic factors using the Cox regression method. They finish this chapter with a discussion on meta-analysis.

The last chapter is headed summary and conclusions. It briefly summarizes their key points on protocol lead randomized clinical trials. It also contains the mission statement of the SWOG statistical centre.

The reader may feel that groups organizing multicentre clinical trials are being overly prescriptive in their demands on the medical collaborators who are actually entering patients. An addendum to their mission statement on how others may see the work of a trials centre interfering with getting results is included, statisticians like to be unbiased! Hopefully, this book instructs the reader on why randomized trials are needed and why they need to be well organized. A final remark is that all proceeds from this book will go to cancer research.

*Rick Swindell*

### IL-12: Chemical Immunology series Vol. 68

Edited by L Adorini: Publication details, pp. 204, £123.90, ISBN 3–8055–6526–7. Karger: Basle, 1997

It seems quite amazing that only a few years ago a single textbook could cover most of the information known about all characterized cytokines, whereas now we have textbooks such as this devoted to a single cytokine. There can be little doubt, however, that IL-12, with its central role in the Th1/Th2 paradigm, does justify such a volume.

The book, in common with most other multiauthor books, does show considerable variation in style with regard to content and emphasis of the individual chapters. There are ten chapters in total. The first four chapters are fairly coherent, concentrating on structural and functional aspects of the cytokine and receptor complex in the human and mouse, and by the end of these chapters the reader has the impression of a good comprehensive review of the subject.

The next chapter is concerned with the modulation of IL-12 activities by other cytokines, such as TGF-β, IFN-γ and IL-4. This chapter is based on experimental results from the authors' laboratory with an extended discussion of these results in the context of other published data. There is no acknowledgement in the chapter that the data presented have been published elsewhere, and one must question the suitability of a largely review book such as this including data that have not been published in a peer-reviewed journal. This chapter concludes that the development of a Th1- or Th2-dominated immune response depends on the presence and concentration of IL-12 and IFN-γ or IL-4 and TGF-β, respectively, early in the immune response.

There are then three chapters devoted to the role of IL-12 in infectious diseases. These are largely concerned with a few organisms, such as *Leishmania*, *Candida albicans*, *Toxoplasma gondii* and *Trypanasoma cruzi*. The first of this group of chapters is also more of a paper than a review, with inclusion of experimental data. The authors' data indicate that, contrary to other published data, IL-4-deficient BALB/c mice can contain *Leishmania* infection unlike the disease susceptible wild-type BALB/c. They conclude that, while IL-4 is not the only factor determining susceptibility, it does tip the balance towards a worse outcome. Again, there is no indication that these data have been reported in any peer-reviewed literature. This chapter is followed by a philosophical discussion of the role of IL-12 in bridging innate and adaptive responses with particular reference to *Candida albicans* and makes interesting reading. The final chapter in this group considers protozoal infections; there is inclusion of some experimental data, but this chapter predominantly reviews the field. It concludes that glycolipids from intracellular protozoa are of importance in inducing IL-12 and other proinflammatory cytokines from macrophages, providing an explanation for the predominant Th1 response to these organisms.

The penultimate chapter would be of most interest to cancer specialists and reviews the animal and human clinical trials exploring the anti-tumour potential of IL-12. This is a comprehensive review, but one has a feeling of déjà vu while reading this chapter. With promising results in animal and in vitro models, phase II human trials were started and then prematurely abandoned because of intolerable adverse side-effects such as gastrointestinal bleeding, asthenia and hepatotoxicity. New approaches using gene therapy are under way, but at the time of preparation of the book it was too early to assess the efficacy of these. This is a very similar story to that for many of the other cytokines investigated in earlier trials, e.g. TNF-α.

The final chapter is again a comprehensive review of IL-12 and Th1/Th2 responses in autoimmune diseases with discussion of the potential for manipulation of Th1/Th2 responses as a therapeutic approach to control of disease induction and progression.

 *© Cancer Research Campaign 1999*

This book would have most appeal for experimental immunologists working in the Th1/Th2 field. It represents a comprehensive review of IL-12 up to the publication date and is very well referenced throughout. However, while the tumour section is well written and relevant it constitutes a relatively small section of the book.

*Professor P Riches and Dr TA Poulton*,
*Department of Immunology*,
*St George's Hospital Medical School*,
*London SW17 0RE*

© Cancer Research Campaign 1999

